# An evaluation tool for Myofascial Adhesions in Patients after Breast Cancer (MAP-BC evaluation tool): Concurrent, face and content validity

**DOI:** 10.1371/journal.pone.0193915

**Published:** 2018-03-09

**Authors:** An De Groef, Marijke Van Kampen, Peter Moortgat, Mieke Anthonissen, Eric Van den Kerckhove, Marie-Rose Christiaens, Patrick Neven, Inge Geraerts, Nele Devoogdt

**Affiliations:** 1 KU Leuven–University of Leuven, Department of Rehabilitation Sciences; University Hospitals Leuven, Department of Physical Medicine and Rehabilitation, Leuven, Belgium; 2 Oscare, Organisation for Burns, Scar After-care and Research, Antwerp, Belgium; 3 Maastricht University Medical Centre, Department of Plastic Surgery, Maastricht, The Netherlands; 4 University Hospitals Leuven, Multidisciplinary Breast Center, Leuven, Belgium; 5 KU Leuven–University of Leuven, Department of Surgical Oncology, Leuven, Belgium; 6 University Hospitals Leuven, Department of Obstetrics and Gynecology, Leuven, Belgium; 7 University Hospitals Leuven, Department of Vascular Surgery, Leuven, Belgium; University of California San Francisco, UNITED STATES

## Abstract

**Purpose:**

To investigate the concurrent, face and content validity of an evaluation tool for Myofascial Adhesions in Patients after Breast Cancer (MAP-BC evaluation tool).

**Methods:**

1) Concurrent validity of the MAP-BC evaluation tool was investigated by exploring correlations (Spearman’s rank Correlation Coefficient) between the subjective scores (0 –no adhesions to 3 –very strong adhesions) of the skin level using the MAP-BC evaluation tool and objective elasticity parameters (maximal skin extension and gross elasticity) generated by the Cutometer Dual MPA 580. Nine different examination points on and around the mastectomy scar were evaluated. 2) Face and content validity were explored by questioning therapists experienced with myofascial therapy in breast cancer patients about the comprehensibility and comprehensiveness of the MAP-BC evaluation tool.

**Results:**

1) Only three meaningful correlations were found on the mastectomy scar. For the most lateral examination point on the mastectomy scar a moderate negative correlation (-0.44, p = 0.01) with the maximal skin extension and a moderate positive correlation with the resistance versus ability of returning or ‘gross elasticity’ (0.42, p = 0.02) were found. For the middle point on the mastectomy scar an almost moderate positive correlation with gross elasticity was found as well (0.38, p = 0.04) 2) Content and face validity have been found to be good. Eighty-nine percent of the respondent found the instructions understandable and 98% found the scoring system obvious. Thirty-seven percent of the therapists suggested to add the possibility to evaluate additional anatomical locations in case of reconstructive and/or bilateral surgery.

**Conclusions:**

The MAP-BC evaluation tool for myofascial adhesions in breast cancer patients has good face and content validity. Evidence for good concurrent validity of the skin level was found only on the mastectomy scar itself.

## Introduction

Awareness of the contribution of myofascial dysfunctions to pain after breast cancer treatment has increased. [1, 2] Torres Lacomba et al reported a prevalence rate of the myofascial pain syndrome of 45% one year after breast cancer surgery. [3] Both myofascial trigger points and adhesions and/or restrictions between the myofascial tissues layers can contribute to a patient’s pain complaint.[3–7]

For myofascial trigger points, several criteria to determine their presence have already been postulated.[3, 8, 9] For the evaluation of myofascial adhesions, the Myofascial Adhesions in Patients after Breast Cancer (MAP-BC) evaluation tool has been developed by De Groef et al.[10] This tool evaluates the degree of myofascial adhesions at 7 anatomical locations (axillary and breast region scars, pectoral muscles region, axilla, frontal chest wall, lateral chest wall and inframammary fold) in breast cancer patients. At each location, the degree of myofascial adhesions is scored at three levels of depth (skin, superficial and deep level) on a 4-points scale (between no adhesions and very strong adhesions). Additionally, a total score between 0 and 9 is calculated, i.e. the sum of the different levels of each location.

The Cosmin Checklist distinguishes three domains in assessing the quality of a measurement instrument, i.e. reliability, validity and responsiveness.[11] The reliability of the MAP-BC evaluation tool has already been investigated.[10] Interrater agreement of the different levels separately was moderate to good (Weighted Kappa (WK) 0.62 to 0.90) for the scars. For almost all levels of the non-scar locations moderate agreement was reached (Weighed Kappa > 0.50), except for 2 levels (superficial level of the lateral chest wall and skin level of the inframammary fold) were only poor agreement was found (WK 0.41 to 0.44). Interrater reliability of the total scores was very good for the scars as well (Intraclass Correlation Coefficient (ICC) 0.82 to 0.99). At non-scar locations, good interrater reliability was reached (ICC 0.76–0.87), except for the inframammary fold (ICC 0.71).

The validity of the MAP-BC evaluation tool has not yet been investigated. Firstly, concurrent validity should be explored by comparing the subjective scores of the MAP-BC evaluation tool to another objective measurement tool. However, to our knowledge, no objective measurement tool or gold standard for the evaluation of the degree of myofascial adhesions in breast cancer patients exists. Nevertheless, several objective measurement tools exist to measure elasticity or stiffness of scars in other populations.[12] The most widely used evaluation tool is the Cutometer Dual MPA 580 Skin Elasticity Meter.[12] The intra- and interrater reliability of the Cutometer has been found to be good to excellent in normal skin and less severe hypertrophic burn scars.[13–15] Reliability in hypertrophic scars is only moderate.[13, 14] In the study of Draaijers et al objective measurements with the Cutometer had moderate and statistically significant correlations with subjective palpation of the pliability of burn scars.[15]

The first aim of this study was to explore the concurrent validity of the MAP-BC evaluation tool by comparing its subjective scores with objective elasticity parameters generated by the Cutometer. Second, face and content validity were explored by questioning physical therapists experienced with myofascial therapy in breast cancer patients. For concurrent validity, we hypothesize 1) a negative correlation for the maximal skin extension and 2) a positive correlation for gross elasticity, both measured with the Cutometer, on the one hand with the degree of myofascial adhesions at the skin level measured with the MAP-BC evaluation tool on the other hand. Second, face and content validity should be explored by questioning other persons with expertise in this domain. With the validation of the MAP-BC evaluation tool better insight in how to evaluate soft tissue restrictions in daily clinical practice will be provided.

## Methods

This study was approved by the Ethical Committee of the University Hospitals of Leuven (s54579). All participants (breast cancer patients and medical workers) gave written informed consent to participate.

### Part 1: Concurrent validity

#### Participants

A convenience sample of 30 women treated for breast cancer were recruited at the Multidisciplinary Breast Center of the University Hospitals of Leuven. Inclusion criteria were 1) unilateral modified radical mastectomy as treatment for breast cancer; 2) the mastectomy scar had to be healed. Patients with complications such as lymphedema and seroma were excluded.

#### MAP-BC evaluation tool

The MAP-BC evaluation tool was used for the subjective assessment of myofascial adhesions by palpation. The degree of adhesions was scored at three depth levels (skin, superficial and deep) and each time on a 4-points scale (between 0—no adhesion and 3—very strong adhesions). Additionally, a total score with a maximum score of 9 was calculated, i.e. the sum of the different levels of each location. More details and instructions on how to use the MAP-BC evaluation tool are reported elsewhere.[10]

#### Cutometer

**Fig 1:**
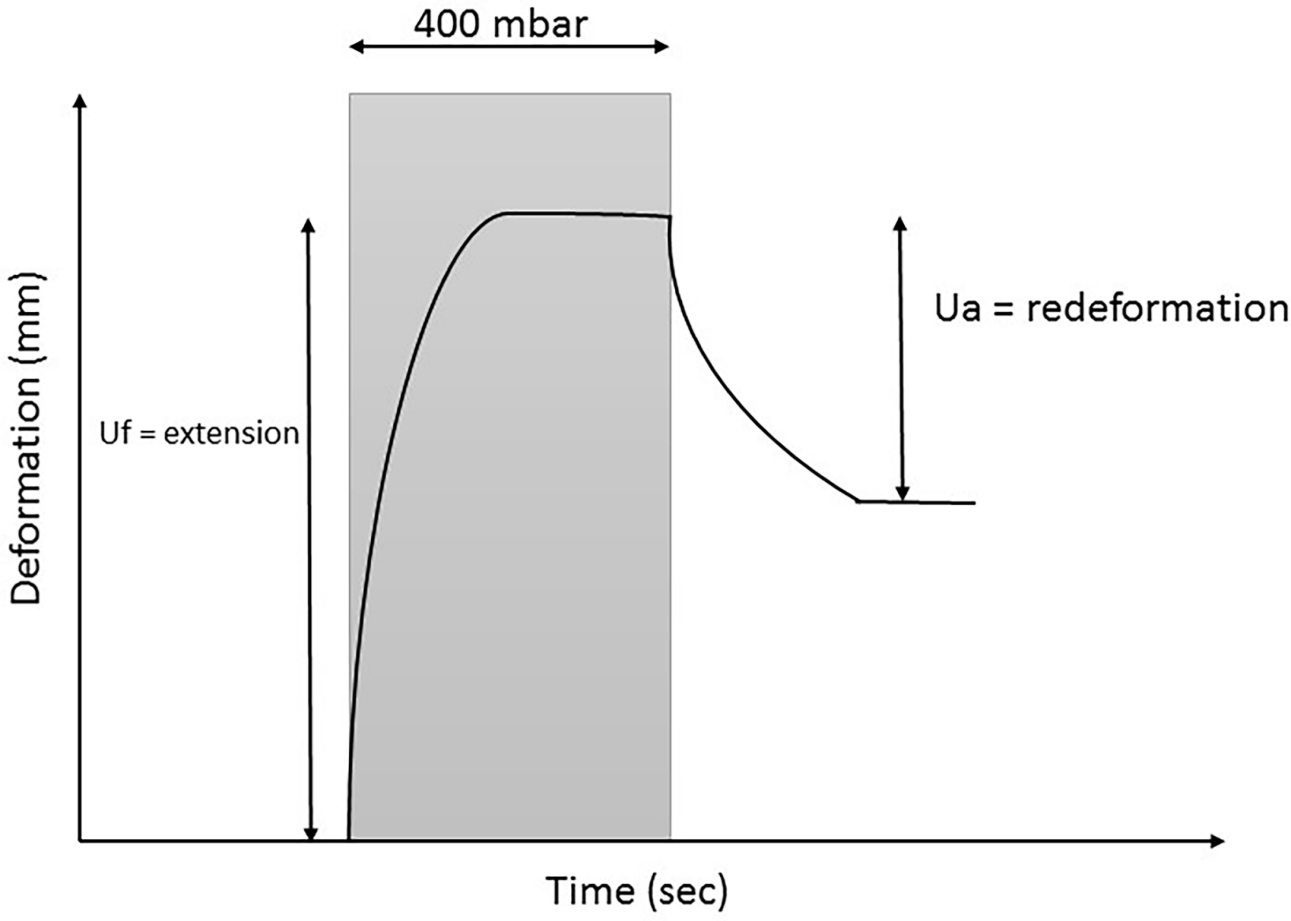
Time-deformation curve of the Cutometer.

The Cutometer Dual MPA 580 (Courage and Khazaka Electronic GmbH, Cologne, Germany) measures the elasticity of the skin using negative pressure which deforms the skin mechanically. The Cutometer measures the vertical deformation of the skin in millimeters when the skin is pulled into the circular aperture (6 mm in diameter and 2 mm in height) of the probe with an exactly defined vacuum pressure. In this study, a vacuum load of 400 mbar was used and suction and relaxation time of 2 seconds. Fig 1 illustrates the deformation-time curve of the Cutometer. Following parameters were used for analysis: first, the maximal skin extension (Uf) is the highest point of the curve (at the end of the vacuum period). It represents the passive behavior of the skin to a force. This parameter gives an implication of the firmness of the skin and has been shown to be the most reliable parameter on scars.[14, 15] We hypothesized a negative correlation between maximal skin extension and the degree of myofascial adhesions at the skin level measured with the MAP-BC evaluation tool. Second, the gross elasticity (Ua/Uf) is expressed as the ratio between the final retraction (Ua) and the maximal skin extension (Uf), which expresses resistance versus ability of returning. We hypothesized a positive correlation between gross elasticity and the degree of myofascial adhesions at the skin level since resistance versus ability of returning of strong adhesions will approximate toward 1 (100%).[16, 17] In the output of the Cutometer these parameters can be found under R0 and R2, respectively.

#### Procedure

**Fig 2:**
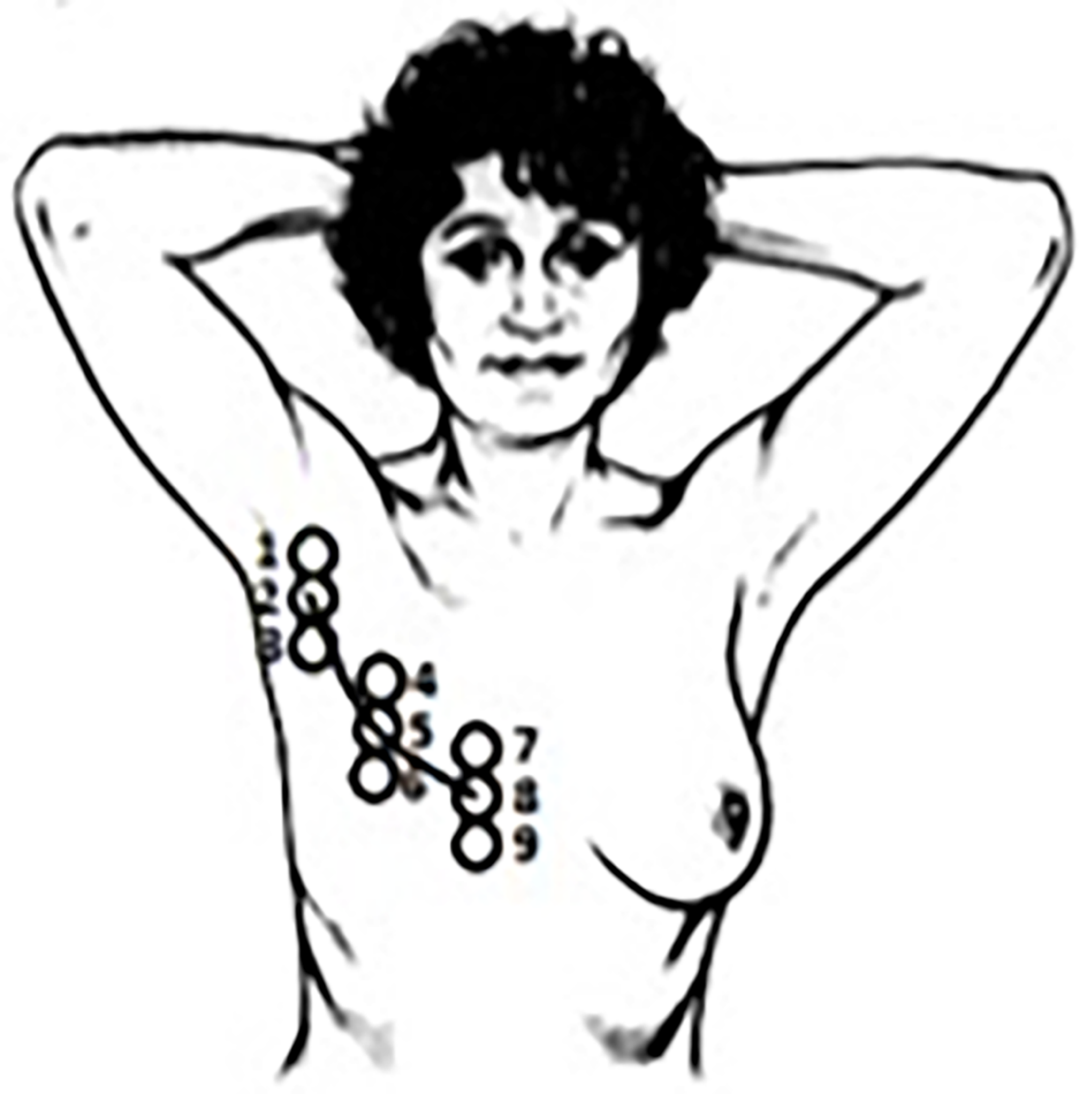
Overview of the nine examination points.

The subjects were asked to lie down in the predetermined position, shown in Fig 2. In case of pain or discomfort, the subject was allowed to place the arms alongside the body.

The evaluation of the elasticity of the scar tissue was limited to nine measuring points on and around the scar (Fig 2) because assessment of the complete upper body is neither feasible nor practical. The measuring points are located at the center and opposite ends of the scar and 2 cm above and below the scar. Measuring points 1, 2 and 3 are situated at the lateral extremity of the scar, near the armpit; measuring points 4, 5 and 6 at the center of the scar and measuring points 7, 8 and 9 at the medial extremity of the scar, near the sternum. The nine measuring points are marked on the skin using a circular template of the probe of the Cutometer device. First, measuring point 2, the lateral end point of the scar, was drawn. The circle was drawn in such a manner that the end point of the scar forms the center of this circle. Subsequently, one circle is drawn two centimeters above and two centimeters below the examination point 2 for point 1 and 3, respectively. This approach was repeated at the opposite extremity of the scar for measuring point 7, 8 and 9. In order to draw the cluster of circles at the center of the scar, the exact center point of the scar was determined by using a measuring tape. Foregoing procedure was repeated for measuring point 4, 5 and 6.

After identifying the measurement points, the rater palpated these nine points and the perceived adhesions at the skin level were scored from 0 to 3 conforming to the MAP-BC evaluation tool. The same rater assessed the degree of adhesion with the Cutometer, in the same order and on equivalent measuring points. The computer screen with the results of the Cutometer was turned away from the rater to maximize blinding for the results of the reference test. Testing was performed by one rater with more than 3 years of experience in treatment of myofascial dysfunctions in breast cancer patients. For the testing with the Cutometer a 2-hour training was implemented.

#### Statistical analysis

The data were analyzed by the statistical software package SPSS 23.0. The concurrent validity was established by means of a Spearman’s rank Correlation Coefficient since data were not normally distributed. Correlations between the scores of the subjective evaluation of the skin level with the MAP-BC evaluation tool and the parameters maximal skin extension (Uf) and gross elasticity (Ua/Uf) measured with the Cutometer were explored. Arbitrary guidelines for interpretation of the correlations are formulated by Evans.[18] A correlation coefficient between 0 and 0.19 indicate a very weak correlation, between 0.20–0.39 weak, between 0.40–0.59 moderate, between 0.60–0.79 strong and between 0.80–1.00 very strong. A convenience sample was recruited without power analysis.

### Part 2: Content and face validity

Face validity is the degree to which the items of the MAP-BC evaluation tool indeed look as though they are an adequate reflection of the construct to be measured.[11] This can be examined by asking the respondent whether the instructions and scoring system of the evaluation tool are understandable and obvious. Content validity is the degree to which the content of the MAP-BC evaluation tool is an adequate reflection of the construct to be measured. This can be examined by analyzing the answers given to the questions about the comprehensiveness of the evaluation tool.

Medical workers, familiar with the application of myofascial therapy in breast cancer patients, were recruited through two educational institutes in Flanders (Belgium) and the Netherlands. The medical workers were asked to complete a questionnaire on face and content validity. The medical workers were recruited through two educational institutes in Flanders (Belgium) and the Netherlands. The questionnaire consisted of 3 questions: 1) ‘Were the instructions of the MAP-BC evaluation tool clear and easy to understand?’, 2) ‘Did you find the scoring system obvious?’ And 3) ‘Did you find the MAP-BC evaluation tool complete?’. Physical therapists who gave a negative answer to any of the questions were asked to provide a more detailed explanation.

In the context of determining the content validity, the relevance of the 7 anatomical locations of the original MAP-BC evaluation tool was explored. The MAP-BC evaluation tool was administered in the 30 participants of Part 1 of this study. First, for each anatomical location the minimum, maximum, median and interquartile range of the degree of myofascial adhesions is given. Second, prevalence rate of myofascial adhesions at each location is given.

## Results

Thirty women after axillary lymph node dissection and modified radical mastectomy for breast cancer were available for testing of concurrent validity. Patients characteristics are given in Table 1. Mean (standard deviation) room temperature and humidity were 23.3° (0.7) and 30.7% (4.4), respectively. Median (interquartile range) total score of the degree of myofascial adhesions at the mastectomy scar measured with the MAP-BC evaluation tool was 4 (3–6). Mean maximal skin extension (Uf) and gross elasticity (Ua/Uf) were 0.79 (0.16) mm and 0.72 (0.06), respectively. The correlation between maximal skin extension (Uf) and gross elasticity (Ua/Uf) was moderate and statistically significant (-0.409, p = 0.025).

**Table 1:** Patients’ characteristics (N = 30). Mean (SD) and Number (%) are given.

Age (years)	53.9 (10.9)
BMI (kg/m^2^)	25.2 (4.0)
Time after surgery (years)	1.2 (0.5)
Chemotherapy	13 (43%)
Neo-adjuvant chemotherapy	11 (37%)
Radiotherapy	30 (100%)
Endocrine therapy	24 (80%)
Target therapy	5 (14%)

N = Number; SD = Standard Deviation; BMI = Body Mass Index

### Part 1: Concurrent validity

An overview of the concurrent validity is given in Table 2. Except for 3 points, no correlations between the subjective evaluation of myofascial adhesion at skin level with the MAP-BC evaluation tool and the objective parameters of the Cutometer were found. All significant correlations were found at measurement points on the mastectomy scar. For the most lateral examination point on the mastectomy scar a moderate negative correlation (-0.442, p = 0.014) with the maximal skin extension was found, meaning the greater the subjective score, the less extension of the scar. At the same point and in the middle of the scar, a moderate positive correlation between the subjective evaluation of the skin level and gross elasticity was found (0.416, p = 0.022 and 0.380, p = 0.038, respectively). These correlations indicated that the higher the subjective score, the greater the ratio between the maximal skin extension and the ability to return or ‘gross elasticity’.

**Table 2:** Concurrent validity.

	Maximal skin extension (Uf)	Gross elasticity (Ua/Uf)
Point 1 –skin	-0.276 (p = 0.140)	0.115 (p = 0.545)
Point 2 –skin	**-0.442 (p = 0.014)**	**0.416 (p = 0.022)**
Point 3 –skin	-0.326 (p = 0.079)	-0.120 (p = 0.528)
Point 4 –skin	-0.333 (p = 0.073)	-0.063 (p = 0.743)
Point 5 –skin	-0.111 (p = 0.558)	**0.380 (p = 0.038)**
Point 6 –skin	-0.106 (p = 0.577)	0.037 (p = 0.844)
Point 7 –skin	0.097 (p = 0.608)	0.292 (p = 0.117)
Point 8 –skin	0.016 (p = 0.932)	0.253 (p = 0.117)
Point 9 –skin	-0.091 (p = 0.631)	-0.061 (p = 0.749)

Uf = maximal skin extension, defined as the highest point of the curve (at the end of the vacuum period); Ua/Uf = Gross elasticity, expressed as the ratio between the final retraction (Ua) and the maximal skin extension (Uf), which expresses resistance versus ability of returning.

### Part 2: Face and content validity

Forty-three medical workers completed the questionnaire on face and content validity. Twenty (47%) of them were physical therapists, 9 (21%) were edema therapists and 14 (32%) were skin therapists. Mean age was 39.1 (10.8) years and mean years of experience in myofascial therapy in breast cancer patients was 4.8 (6.7) years.

Thirty-nine (89%) medical workers found the instructions of the MAP-BC evaluation tool understandable. Two persons suggested to describe the amount of pressure for palpation in the instructions. One person found the wording ‘early restriction’ not clear. Forty-two (98%) medical workers found the scoring system obvious. One person made the remark that distinction between the different depth levels may be difficult in case of very stiff adhesions.

Twenty-seven (63%) medical workers found the MAP-BC evaluation tool complete. Several persons (exact number between parentheses) suggested to add six more anatomical locations: scars after reconstruction (n = 2), the back (n = 5), the arm for the brachial fascia (n = 2), the nipple (n = 1), the abdomen (n = 2) and the platysma or neck region (n = 5). Additionally, two persons indicated they want to be able to report the direction of the myofascial restriction and one person made the remark that the MAP-BC evaluation tool is not suitable for bilateral surgery.

For each anatomical location the minimum, maximum, median and interquartile range of the degree of myofascial adhesions and the prevalence rate of myofascial adhesions are given in Table 3. All patients had to a certain extent myofascial adhesions at the mastectomy scar. Adhesions at the inframammary fold were the least frequent. At all locations, except the lateral chest wall and the inframammary fold, the full range of the scoring system (degree of adhesions 0–3) was used.

**Table 3:** For each anatomical location (7) the minimum, maximum, median and interquartile range of the degree of myofascial adhesions is given. Additionally, prevalence rate of myofascial adhesions at that location is given.

	*Minimum*	*Median (IQR)*	*Maximum*	*Number (%) of patients with adhesions*
**Mm pectoralis region**				
Skin	0	0 (0–1)	2	13 (43%)
Superficial	0	2 (1–2)	3	27 (90%)
Deep	0	2 (1–2)	3	28 (93%)
Total score	0	4 (2–5)	7	29 (97%)
**Axilla**				
Skin	0	1 (0–1)	2	17 (57%)
Superficial	0	1 (1–2)	3	27 (90%)
Deep	0	1 (1–2)	3	25 (83%)
Total score	0	3 (2–5)	8	29 (97%)
**Frontal Chest Wall**				
Skin	0	1 (0.75–2)	2	23 (76%)
Superficial	0	1 (0–2)	3	22 (73%)
Total score	0	2 (1–4)	5	24 (80%)
**Lateral Chest Wall**				
Skin	0	1 (1–1)	2	28 (93%)
Superficial	0	1 (0–2)	2	21 (70%)
Deep	0	1 (0–2)	2	19 (63%)
Total score	0	3 (1–5)	6	29 (97%)
**Inframammary fold**				
Skin	0	1 (0–1)	2	16 (53%)
Superficial	0	0 (0–1.25)	2	14 (47%)
Deep	0	0 (0–1)	2	13 (43%)
Total score	0	1 (0–3.25)	6	21 (70%)
**Axillary scar**				
Skin	0	1 (1–1.25)	2	28 (93%)
Superficial	0	0 (0–1)	2	12 (40%)
Deep	0	0 (0–1)	3	10 (33%)
Total score	0	1 (1–4)	6	29 (97%)
**Mastectomy Scar**				
Skin	1	2 (1–2)	3	18 (60%)
Superficial	0	1 (1–2)	3	26 (87%)
Deep	0	1 (1–2)	3	25 (83%)
Total score	1	4 (3–6)	8	30 (100%)

IQR = Interquartile Range

## Discussion

In the first part of this study the concurrent validity of the MAP-BC evaluation tool was investigated. Nine different examination points on and around the mastectomy scar were evaluated at the skin level. Only three meaningful and significant correlations were found at the lateral end and the middle of the mastectomy scar. The subjective evaluation of the skin level with the MAP-BC evaluation tool was significantly correlated to the maximal skin extension and the resistance versus ability of returning or ‘gross elasticity’ measured with the Cutometer. Additionally, good face and content validity of the MAP-BC were found.

At the most lateral examination point on the mastectomy scar itself, a negative correlation between the subjective evaluation of the skin level of the mastectomy scar and the maximal skin extension measured with the Cutometer was found. This negative correlation indicates that a higher subjective score for myofascial adhesions is correlated to a smaller skin extension generated by the Cutometer. This result is similar to the correlation between the subjective evaluation of pliability of burn scars and maximal skin extension in the study of Draaijers et al.[15] At the same point and in the middle of the scar, a positive correlation between the subjective evaluation of the skin level and gross elasticity was found. This correlation indicates that the higher the subjective score, the greater the resistance versus the ability of returning or ‘gross elasticity’. ‘Gross elasticity’ takes into account two parameters. In strong adhesions, maximal skin extension will be rather small but final retraction or the ability to return will be rather great, approximating a ratio of 1 or 100%. Similar correlations between gross elasticity and deep burn scars[16] or younger skin[17] have been found. Based on only these three significant correlations, we could conclude moderate concurrent validity of the MAP-BC evaluation tool for the mastectomy scar at skin level only.

The Cutometer was chosen as objective measurement tool because of the good reliability and validity[13, 14] and the widely-accepted use as objective tool in the evaluation of skin elasticity in other populations. However, some characteristics of the Cutometer may explain the poor correlations with the MAP-BC evaluation tool. First, only the skin is evaluated with the Cutometer. More specific, only the epidermis and (papillary) dermis are evaluated. Breast and axillary surgery and radiotherapy in breast cancer patients cause scar tissue formation, fibrosis and adhesions at all levels of soft tissues of the upper body. Therefore, the MAP-BC was designed to evaluate the skin level and deeper myofascial structures. Second, the Cutometer produces a vertical deformation of the skin while the palpation is more in the horizontal plane. Third, the Cutometer has not been used before in breast cancer patients. Scar tissue formation in burns or skin grafts may be too different from the fibrosis, adhesions and scar tissue formation in breast cancer patients. In burns or skin grafts, typical characteristics are an aberrant color, rough surface texture and/or increased thickness (i.e. hypertrophy).[19] In breast cancer patients, radiotherapy-induced fibrosis, linear scars from breast and axillary surgery and internal scar tissue formation in the surgical area may be present. For example, during the mastectomy procedure the glandular tissue is removed and the skin is folded towards the thorax. Additionally, drains are inserted after surgery. These internal wounds heal by forming scar tissue and adhesions which are of a different type compared to burn scars. Therefore, it is possible that certainly for measurement points around the mastectomy scar, the Cutometer measures a different construct than the MAP-BC evaluation tool whereby no meaningful correlations were found. Fourth, skin elasticity measurements with the Cutometer are localized at a very small surface of 6 mm diameter and measurements are sensitive to location changes.[13] The MAP-BC evaluation tool measures a larger surface area. Possibly, the (small) differences in skin elasticity in the larger surface are not detectable by palpation and the score of the MAP-BC evaluation tool represents a mean score of the larger surface.

Other objective evaluation tools to measure scar elasticity do exist but have their limitations as well.[12] First, comparable to the Cutometer is the DermaLab Suction Cup. Reliability has been found to be good in normal skin, burn scars and fibrotic skin.[20, 21] However, this device measures the force necessary to lift the skin a certain height and therefore may be limited in use in very stiff scars or adhesions. Second, pressure methods or ‘tonometers’ are not useful in the breast cancer population because they are not applicable above bone structures (e.g. ribs).[12, 19, 21] Third, torsion methods may be suitable since they use a horizontal deformation force more likely to the palpation method in the MAP-BC evaluation tool. However, usefulness of the torsion method in scar assessment has not yet been investigated. Only one study has used this method in the evaluation of skin grafts.[22] Fourth, extension methods or ‘extensometers’ stretch the skin between two tabs to assess differences in extensibility or stiffness. This may be useful to compare with the MAP-BC, however this is again a vertical deformation and to our knowledge scientific results are limited.[12]

In the second part of this study, face and content validity were investigated. The instructions were understandable and the scoring system was obvious for almost all medical workers. The comprehensibility of the questionnaire was very good. The remark on the description of the amount of pressure for palpation of the different depth levels was given by 2 persons with only 2 and 3 months experience in myofascial therapy, respectively. For therapists with only little experience in the evaluation and treatment of myofascial adhesions a more elaborated description on the palpation techniques may indeed be useful. For the skin level the amount of pressure can be compared to the pressure on an eyelid. For the other depth levels, an adequate description of the amount of pressure is more complicated. Another person indicated that the wording ‘early restriction’ was not clear.

Most remarks were made on the completeness of the MAP-BC evaluation tool. The authors included the most frequent affected anatomical locations after unilateral mastectomy or breast conserving surgery, based on expert opinions and literature. In case of bilateral surgery and/or reconstructive surgery it may indeed be useful to examine the back (in case of e.g. latissimus dorsi flap reconstruction), the abdomen (in case of e.g. Deep Inferior Epigastric Perforators flap) and additional scars. Therefore, the authors suggest adding an ‘empty’ illustration on the MAP-BC evaluation tool that can be used for additional anatomical regions, if necessary. This additional illustration can also be used, for example, at the nipple, the arm, the platysma and the neck as suggested. Currently, the direction of the myofascial restriction is not incorporated in the scoring system. However, it may be clinically relevant to draw the direction of the restriction on the illustration. At last, content validity is illustrated by the variability of the MAP-BC in this representative sample of breast cancer patients. From the prevalence rate of myofascial adhesions, it appears that all anatomical locations are relevant. The scars are the most relevant and the inframammary fold the least. At all locations, a minimum total score was noticed and at five out of seven locations the maximum total score was reached. These results add to the good content validity and the relevance of all items of the MAP-BC evaluation tool.

### Strengths and limitations

The measurements were performed in a highly-standardized manner. The environmental factors had small standard deviations indicate they were quite constant over all measurements. Face and content validity were investigated in a substantially large (N = 43) group of therapists experienced in myofascial therapy. Due to practical reasons and limitations of the Cutometer it was not possible to examine other anatomical locations of the MAP-BC evaluation tool. And finally, no power calculation was performed to determine sample size.

### Future research

Future research should explore the usefulness of the above-mentioned objective instruments for the evaluation of myofascial adhesions in breast cancer patients. Additionally, the utility of ultrasound elastography for the evaluation of soft tissue elasticity and stiffness should be explored. More and more research has been done on the use of different kinds of ultrasound elastography for the evaluation of mechanical properties of tissue, including muscle stiffness, with promising results.[23] Possibly, the construct measured with ultrasound elastography leans more towards the construct measured with the MAP-BC evaluation tool. Future research should explore the correlation between these two evaluation tools. In addition to reliability and validity, responsiveness of the MAP-BC evaluation tool should be explored.[11]

The MAP-BC evaluation tool shows very good face and content validity, indicating the tool is useful in clinical practice. The authors suggest adding an additional blank illustration for the evaluation of additional myofascial adhesions at other anatomical locations. Identifying the exact location and degree of adhesions is essential for steering treatment. The MAP-BC evaluation tool is developed specific for scars related to (breast) cancer treatments. Traditional scar tissue evaluation tools such as the Patient and Observer Scar Assessment Scale (POSAS) are hard to apply in the cancer population because the different types of scar tissue. The MAP-BC may be crucial to fill this gap in daily clinical practice.

### Conclusion

Based on only three significant correlations, we could conclude moderate concurrent validity of the MAP-BC evaluation tool for the mastectomy scar itself. Possibly, other objective measurement tools are better suited to explore the concurrent validity of the MAP-BC evaluation tool. Face and content validity have been found to be good.
